# Nonpharmacological Strategies to Prevent Contrast-Induced Acute Kidney Injury

**DOI:** 10.1155/2014/463608

**Published:** 2014-03-26

**Authors:** Paweena Susantitaphong, Somchai Eiam-Ong

**Affiliations:** ^1^Division of Nephrology, Department of Medicine, Faculty of Medicine, Chulalongkorn University, King Chulalongkorn Memorial Hospital, Bangkok 10330, Thailand; ^2^Extracorporeal Multiorgan Support Dialysis Center, Division of Nephrology, Department of Medicine, King Chulalongkorn Memorial Hospital, Faculty of Medicine, Chulalongkorn University, Bangkok 10330, Thailand

## Abstract

Contrast-induced AKI (CI-AKI) has been one of the leading causes for hospital-acquired AKI and is associated with independent risk for adverse clinical outcomes including morbidity and mortality. The aim of this review is to provide a brief summary of the studies that focus on nonpharmacological strategies to prevent CI-AKI, including routine identification of at-risk patients, use of appropriate hydration regimens, withdrawal of nephrotoxic drugs, selection of low-osmolar contrast media or isoosmolar contrast media, and using the minimum volume of contrast media as possible. There is no need to schedule dialysis in relation to injection of contrast media or injection of contrast agent in relation to dialysis program. Hemodialysis cannot protect the poorly functioning kidney against CI-AKI.

## 1. Introduction 

The definition of acute kidney injury (AKI) is reclassified according to the Kidney Disease Improving Global Outcomes (KDIGO) staging system. Using the KDIGO definition, 1 in 5 adults and 1 in 3 children worldwide experience AKI during a hospital episode of care [1]. Contrast-induced AKI (CI-AKI) is defined as a ≥0.5 mg/dL rise or a 25% increase in serum creatinine, assessed within 48–72 hours after administration of contrast media. CI-AKI is one of the most common causes of hospital-acquired AKI. The incidence varies from less than 2% in general population up to 50% in patients with advanced kidney disease [2]. A recent meta-analysis regarding incidence of CI-AKI after contrast-enhanced computed tomography (CT) showed that the pooled incidence of CI-AKI was 6.4% (95% CI 5.0–8.1). The risk of renal replacement therapy requirement after CI-AKI was low (0.06% 95% CI 0.01–0.4). The decline in renal function persisted in 1.1% of patients (95% CI 0.6–2.1%). Patients with chronic kidney disease (CKD) (odds ratio 2.26, *P* < 0.001) or diabetes mellitus (odds ratio 3.10, *P* < 0.001) were at increased risk for the development of CI-AKI [3]. CI-AKI is also associated with an increased risk of mortality, cardiovascular events, CKD, and prolonged hospitalization [4].

The number of published studies on CI-AKI has dramatically increased during the past decade. Since CI-AKI is a potentially preventable clinical condition, the more the knowledge regarding CI-AKI is understood, the greater the likelihood of reducing the risk. The aim of this review is to provide a brief review and summary of the studies that focus on nonpharmacological strategies to prevent CI-AKI.

## 2. Pathophysiology of CI-AKI

Besides direct tubular toxicity and intraluminal obstruction, renal hypoxia and concomitant release of reactive oxygen species (ROS) have also been considered as important mechanisms of renal injury in CI-AKI [5, 6]. Hypoxia results from the imbalance of oxygen supply from renal blood flow and oxygen demand from renal tissue. Impaired oxygen supply is caused by the reduction of effective renal cortical-medullary blood flow and afferent arteriole constriction mediated via the tubuloglomerular feedback from osmotic diuresis [7].

In addition, hyperosmotic contrast media also cause diuresis and natriuresis that stimulate the macula densa to release adenosine for the activation of adenosine A1 receptors, resulting in vasoconstriction of the afferent arteriole of the glomerulus as well as the medullary vascular bed [6]. Regarding oxygen demand, the increased reabsorption from osmotic load could accelerate metabolism and results in heightened oxygen consumption in the kidney, finally leading to the microvascular damage and intrarenal hypoxia [7].

Furthermore, renal medullary hypoxia may also produce ROS to scavenge nitric oxide (NO). Superoxide (O_2_^−^), hydrogen peroxide (H_2_O_2_), and hydroxyl radical (OH^−^) are the most common ROS [6]. Moreover, the production of these ROS could be aggravated by oxidative stress in the mitochondria [6]. Increased markers of ROS were evident in the urine and plasma of patients after cardiac catheterization, particularly in those with CKD and diabetes mellitus [8].

## 3. Nonpharmacological Strategies to Prevent CI-AKI

### 3.1. Evaluation of the Risk of CI-AKI and Consideration of Alternative Imaging Methods

All patients going to receive contrast media should be evaluated for the risk of CI-AKI. Prophylaxis with therapies that are supported by clinical evidence should be considered in high-risk patients. If it is possible, alternative imaging methods without contrast media in high-risk patients should be performed.

Moos et al. [9] summarized the incidence of CI-AKI and the associations between CI-AKI incidence and risk factors in patients undergoing intravenous contrast-enhanced CT with iodinated low- or isoosmolar contrast media (LOCM and IOCM, resp.). The authors reported that the overall pooled CI-AKI incidence was 4.96% (95% CI: 3.79–6.47) and found the significant associations between CI-AKI and those who had renal insufficiency, diabetes mellitus, and malignancy, are of age > 65 years, and used nonsteroidal anti-inflammatory drugs (NSAIDs). Moreover, despite having a normal baseline creatinine, diabetic patients are at an increased risk of developing CI-AKI, particularly older patients and patients with high urine albumin/creatinine ratio [10].

Serum uric acid level [11], neutrophil-to-lymphocyte ratio as a systemic inflammation marker [12], pulse pressure [13], aortic stiffness represented as pulse wave velocity and augmentation index [14], and anemia [15, 16] are simple independent early predictors of CI-AKI in patients who were exposed to contrast media and are probably used for early detection that may attenuate the progression of CI-AKI.

Gurm et al. [17] also proposed the model for predicting CI-AKI including patient's conditions such as the presence of heart failure and cardiogenic shock as well as the values of laboratory setting such as hemoglobin and creatinine (area under the receiver-operating characteristic curve (AUC) for predicting CI-AKI = 0.85). The details of this model can be downloaded from the website https://bmc2.org/calculators/cin.

Recently, the Contrast Media Safety Committee of European Society of Urogenital Radiology (ESUR) has reported the risk factors for CI-AKI (Table 1) [18]. 

*Electronic Warning Systems.* Cho et al. [19] demonstrated the benefit of using computerized alertness program in hospitalized patients. When contrast-enhanced CT was ordered in patients with a GFR of <60 mL/min/1.73 m^2^, the physician was immediately alerted by a warning message to consider prophylactic measures for CI-AKI. This electronic warning systems significantly decreased the risk of CI-AKI (3% versus 10%, *P* = 0.02) [19].

### 3.2. Drug Review and Medication Discontinuation

Prior to contrast media exposure, the use of established nephrotoxic drugs, for example, cyclosporine A, aminoglycoside, and NSAID including COX-2 inhibitors, should be stopped for at least 2 days. Diabetic patients with preexisting renal impairment should withhold metformin for 48 hours because lactic acidosis may occur once CI-AKI develops. However, patients with normal renal function who are taking metformin are not at risk of CI-AKI and should be assessed according to their overall clinical conditions [20].

The pathogenic role of angiotensin-converting enzyme (ACE) inhibitors in CI-AKI is still controversial. Some studies pointed out that ACE inhibitors were effective in the prevention of CI-AKI, while some concluded that they were associated with increased risk of CI-AKI, especially for patients with preexisting renal impairment. On one hand, experimental data suggest that activated renin-angiotensin-aldosterone system, enhanced endothelin-1, and increased ROS play important roles in the pathogenesis of CI-AKI and these can be inhibited by ACE inhibitors. On the other hand, ACE inhibitors impair angiotensin II synthesis. In the presence of contrast-induced acute reduction of renal blood flow, blunting the vasoconstriction of efferent arterioles by decreased angiotensin II levels may have a deleterious effect on GFR via decreasing the intraglomerular pressure. These opposite effects probably explain why the studies to date have provided inconclusive results on this issue [21].

Kwok et al. [22] performed systematic review and meta-analysis involving 9 different interventions for CI-AKI; furosemide was shown to increase the risk of CI-AKI (RR 3.27, 95% CI 1.48–7.26). Therefore, this drug should be withheld for avoiding dehydration status before receiving contrast media. However, in patients who still had volume overload such as congestive heart failure or pulmonary edema, furosemide should be used to establish normovolemia prior to contrast exposure.

Lapi et al. [23] reported that current use of a double therapy combination of either diuretics or ACE inhibitors or angiotensin receptor blockers with NSAIDs was not associated with an increased rate of CI-AKI. On the contrary, the current use of a triple therapy combination was correlated with an augmented rate of CI-AKI (rate ratio 1.31, 95% confidence interval 1.12 to 1.53). The highest risk was observed in the first 30 days of use (rate ratio 1.82, 95% confidence interval 1.35 to 2.46).

### 3.3. Volume Expansion

All patients receiving contrast media should have an optimal volume status at the time of exposure. Indeed, volume supplementation plays an important role in the prevention of CI-AKI via two mechanisms. First, expansion of the intravascular space is thought to blunt the vasoconstrictive effect of contrast on the renal medulla via suppression of vasopressin secretion, inhibition of the renin-angiotensin axis, and increased synthesis of vasodilatory renal prostaglandins. Second, intravenous fluid replacement is believed to attenuate the direct toxic effect of contrast agents on tubular epithelial cells by decreasing the concentration and viscosity of contrast media in the tubular lumen. This attenuating effect is the result of volume-mediated inhibition of proximal tubular salt and water reabsorption and decreasing contact time from the associated increase in tubular flow [24].

In patients without heart failure, parenteral isotonic normal saline (0.9% NaCl) without any diuretics should be started 12 hours prior to contrast media administration with an infusion rate of 1 mL/kg/hour and continued for 24 hours. The use of sodium bicarbonate (NaHCO_3_) infusion may not only allow for shorter period of volume supplementation but also further reduce the generation of injurious ROS. Typically, patients should receive 154 mEq/L of NaHCO_3_, as a bolus of 3 mL/kg/hour for 1 hour prior to contrast media administration, followed by an infusion of 1 mL/kg/hour for 6 hours after the procedure [25].

An earlier meta-analysis could not demonstrate the superior benefit of NaHCO_3_ when compared with normal saline [26]. Recent large meta-analysis studies revealed that NaHCO_3_ had a greater benefit than sodium chloride in terms of a change in serum creatinine but provided no significant differences in the occurrence of death and requirement for renal replacement therapy which were of much more important concerns [27–30]. Subgroup analysis by the type of contrast media was performed and suggested lower odds of CI-AKI with NaHCO_3_ in studies using LOCM (OR 0.40; 95% CI 0.23–0.71, *P* = 0.002) compared with IOCM (OR 0.76; 95% CI 0.41–1.43; *P* = 0.40) [30]. The relatively low quality of the individual study, heterogeneity, and possible publication bias mean that only a limited recommendation can be made in favor of the use of NaHCO_3_ (Table 2).

Surprisingly, a recent meta-analysis [31] reported that the oral route of volume expansion may be as effective as the intravenous route for volume expansion for CI-AKI prevention (odds ratio 1.19; 95% CI 0.46, 3.10, *P* = 0.73). In addition, Marenzi et al. [32] also demonstrated that furosemide-induced high urine output with matched hydration (receiving an initial 250 mL intravenous bolus of normal saline over 30 min followed by an intravenous bolus 0.5 mg/kg of furosemide) significantly reduced the risk of CI-AKI and might be associated with improved in-hospital outcome. Hydration infusion rate was automatically adjusted to precisely replace the patient's urine output. When a urine output rate > 300 mL/hour was obtained, patients underwent the coronary procedure. Matched fluid replacement was maintained during the procedure and for 4 h after treatment.

### 3.4. Contrast Agent

#### 3.4.1. Type of Contrast Agent (Table 3)

In a meta-analysis of 25 trials, the pooled odds ratio of CI-AKI with LOCM was 0.61 (95% confidence interval (CI), 0.48–0.77) times that after high osmolar contrast media (HOCM). For CKD patients, this odds ratio was 0.5 (95% CI, 0.36–0.68), while it was 0.75 (95% CI, 0.52–1.1) in patients without prior renal insufficiency. These data suggest that HOCM is generally more nephrotoxic than LOCM and the use of LOCM may be beneficial, particularly in CKD patients [33]. Furthermore, a meta-analysis from 16 double-blind, randomized, controlled trials demonstrated significantly decreased risk of CI-AKI associated with the use of IOCM compared with LOCM, especially in patients with CKD or CKD and diabetes mellitus [34].

However, the risk of CI-AKI with two LOCM including iohexol and ioxaglate was significantly higher than other LOCM (for example, iopamidol, iopromide, and ioversol) and the IOCM (iodixanol) in many studies [35, 36]. In addition, iopamidol and iodixanol are preferable to the others because both reduce the risk of CI-AKI and are less costly and appear to be cost-effective when compared with iohexol or other LOCM [37].

Hence, the CI-AKI Consensus Working Panel suggests that, for intra-arterial administration in high-risk patients with CKD, particularly those with diabetes mellitus, nonionic IOCM are associated with the lowest risk of CI-AKI [38]. In addition, the current guidelines of the American College of Cardiology/American Heart Association recommend the use of either IOCM or LOCM other than iohexol and ioxaglate in CKD patients undergoing angiography [39]. Either IOCM or LOCM, except iohexol or ioxaglate, can be used in all patients. IOCM (iodixanol) may be a better choice for high-risk patients with CKD requiring intra-arterial administration.

#### 3.4.2. Temperature

The contrast media should be prewarmed to 37°C for decreasing viscosity (Table 3).

#### 3.4.3. Route of Administration

In a previous study, the incidences of AKI after intra-arterial and intravenous contrast media administrations were comparable [40]; however, IOCM (iodixanol) significantly decreased the risk of CI-AKI (risk ratio (RR) = 0.68; 95% CI 0.50–0.92; *P* = 0.01) with intra-arterial route, but not with intravenous application (RR = 0.75; 95% CI 0.44–1.26; *P* = 0.27), when compared with a pool of LOCM in a recent meta-analysis [41].

#### 3.4.4. Contrast Volume and Multiple Studies

Multivariate analyses have established the correlation between higher dose of contrast media and risk for CI-AKI [42]. The CI-AKI Consensus Working Panel concludes that the higher contrast volumes (>100 mL) are associated with the higher rates of CI-AKI in patients at risk [38].

Tan et al. [43] proposed the usage of the value derived from contrast media volume divided by creatinine clearance (V/CrCl) above 2.62 for predicting the risk for CI-AKI.

*Automated Contrast Injector Systems (ACIS)*. Contrast volume is a major modifiable risk factor for CI-AKI. ACIS are believed to be associated with a reduction in the total volume of contrast media. Unfortunately, the use of ACIS was associated with a statistically significant lower in the average volume of contrast media with no difference in the incidence of CI-AKI or new need for dialysis [44].

In a recent meta-analysis in 79,694 patients from 10 studies, ACIS reduced contrast volume delivery by 45 mL/case (*P* < 0.001, 95% CI −54 to −35). The CI-AKI incidence was significantly reduced by 15% with an odds ratio of 0.85 (*P* < 0.001, 95% CI 0.78 to 0.93) for those using ACIS compared with manual injection [45]. In addition, a significant association between contrast media dose increment and high prevalence of CI-AKI was demonstrated in a recent cohort [46]. Therefore, using the lowest dose of contrast media should be emphasized.

In terms of the time interval between procedures that require intravascular contrast media administration, the Contrast Media Safety Committee of ESUR recommends that the ideally optimal time interval should be more than 2 weeks which are expected for recovery time of the kidney after acute injury from contrast media. When this is not possible, the interval should be as long as clinically acceptable.

In conclusion, the contrast media should be injected at the lowest possible dose. Repeated injection especially within 72 hours should be avoided and may be requested after 2-week period from the first exposure.

### 3.5. Follow-Up Assessment of Kidney Function

Serum creatinine at 48–72 hours following contrast media exposure should be assessed for CI-AKI detection.

### 3.6. Dialysis (Hemodialysis, Hemofiltration, or Peritoneal Dialysis)

Contrast media are excreted mainly by glomerular filtration. Thus, there is a significant correlation between renal clearances of contrast media and glomerular filtration rate. Thus, renal excretion of contrast media will be delayed in CKD patients. A single session of hemodialysis (HD) can effectively remove 60–90% of contrast media from the blood. Because most contrast media are middle-sized molecules, the main factors potentially affecting CI-AKI depend on HD efficacy of contrast media removal. Blood flow, membrane surface area, molecular size, transmembrane pressure, and dialysis time are important factors that contribute to the efficacy of HD [47]. Generally, several hemodialysis sessions are needed to eliminate all contrast media. Some studies have explored the necessity of immediate HD after intravascular injection of contrast media in chronic HD patients; the authors demonstrated no effective evidence for preventing CI-AKI [48]. The reasons why HD treatment was not beneficial in these studies are still unestablished. The rapid onset of renal injury after administration of contrast media might partly explain such finding. However, Marenzi et al. [49] reported the more efficacy of periprocedural hemofiltration in preventing CI-AKI in CKD patients undergoing coronary interventions.

Peritoneal dialysis is also effective in removing contrast agents from the body but takes longer duration time period than HD. Three weeks of continuous ambulatory peritoneal dialysis is needed for completely removing the agent. Contrast media can be removed effectively by various peritoneal dialysis modalities, including intermittent peritoneal dialysis, automated peritoneal dialysis, and continuous ambulatory peritoneal dialysis [47].

A previous meta-analysis [50] and a recent meta-analysis [51] could not demonstrate the benefit of dialysis on the incidence of CI-AKI when compared with routine preventive care. However, in sensitivity analyses, limiting to only HD studies which could significantly reduce heterogeneity among the included studies, HD appeared to increase CI-AKI risk (RR 1.61; 95% CI, 1.13–2.28) but had no effect on the need for permanent RRT or progression to end-stage renal disease (RR 1.47; 95% CI, 0.56–3.89) [51] (Table 4).

Interestingly, Lee et al. [52] demonstrated the benefit of HD after coronary angiogram in patients with CKD stage V with a mean baseline creatinine clearance of 12.9 mL/min/1.73 m^2^. Approximately, 60% had diabetes mellitus and the mean volume of total contrast media (iohexol) was more than 100 mL that contributed to a high risk of CI-AKI. All patients were given intravenous normal saline at 1 mL/kg/hour for 6 hours before and 12 hours after contrast media exposure. HD was prescribed by using a high-flux dialyzer. The blood flow was 150 mL/min, the duration of dialysis was 4 hours, and the dialysate flow was 500 mL/min. To lessen the hemodynamic changes, 200 mL normal saline priming was administered before dialysis and no fluid removal was prescribed in the dialysis group. HD was initiated at an interval of 81 ± 32 min, ranging from 45 to 180 min, after exposure to the contrast media. A potential important limitation of this study is the use of serum creatinine to diagnose CI-AKI. Indeed, any initial reduction of serum creatinine in HD group is likely a falsely beneficial effect resulting from creatinine removal. The bicarbonate dialysate used and additional fluid replacement might be another important effect. However, the prophylactic dialysis seems not to be important even in advanced CKD.

In addition, the risks of dialysis procedures and the much greater cost should be considered. As such, the Contrast Media Safety Committee of ESUR states that there is no need to schedule the dialysis in relation to the injection of contrast media or the injection of contrast agent in relation to the dialysis program. Hemodialysis does not protect the poorly functioning kidney against CI-AKI [53].

### 3.7. Coronary Sinus Contrast Removal System

As a strong relationship between contrast load and incidence of CIN is obviously demonstrated, alternative strategies to limit the systemic contrast exposure are being developed. The CINCOR catheter (Contrast Removal System, Osprey Medical, St. Paul, Minnesota, USA) has been innovated for removing contrast media from coronary sinus shortly after contrast delivery [54]. Contrast media were effectively withdrawn (44 ± 8%) as assessed by fluoroscopy [55]. Early data demonstrated the benefit of the procedure in attenuating CI-AKI compared with the standard care in small cohort studies [56, 57]. However, one limitation of this system was the requirement for 14 French internal jugular vein sheath insertion. A large-scale randomized trial to evaluate the capacity of this device to reduce the risk of CI-AKI and its complications is required.

### 3.8. Oxygenation Support

Sekiguchi et al. [58] randomly allocated 349 eligible patients who underwent elective coronary angiography and/or percutaneous coronary intervention to either an oxygenation group (oxygen administration via nasal cannula; 2 L/min of oxygen from 10 min before the procedure to the end of the procedure; *n* = 174) or to a control group (room air; *n* = 175). Continuous infusion of isotonic saline solution (1 mL/kg/hour) was administered 12 hours before the procedure until 12 hours after the procedure in both groups. The PaO_2_ at the baseline was significantly higher in the oxygen preconditioning group than the control group (134 ± 28 versus 90 ± 12 mm Hg, *P* < 0.001). The authors demonstrated that the oxygen preconditioning reduced the incidence of CI-AKI, particularly in CKD patients, via decreased intrarenal hypoxia. Therefore, this simple strategy to attenuate CI-AKI might be beneficial in CKD patients.

### 3.9. Remote Ischemic Preconditioning (IPC)

Er et al. [59] demonstrated that remote IPC induced by intermittent upper-arm ischemia prior to elective coronary angiography dramatically reduced the incidence of CI-AKI in patients with CKD and those at high risk of CI-AKI (OR 0.21, 95% CI 0.07–0.57, *P* = 0.002). The IPC was performed as 4 cycles of alternating 5-minute inflation and 5-minute deflation of a standard upper-arm blood pressure cuff to the individual's systolic blood pressure plus 50 mm Hg to induce transient and repetitive arm ischemia and reperfusion. Although the protective mechanism of IPC is still unestablished, it is postulated that a remote organ might release humoral factors such as adenosine, bradykinin, or erythropoietin into the systemic circulation, all of which subsequently protect the remote region or organ.

## 4. Conclusion

The Contrast Media Safety Committee of ESUR [18] has updated its guidelines on CI-AKI (Table 5). First, identify high-risk patients, especially those with eGFR < 60 mL/min/1.73 m^2^, diabetes mellitus, recent nephrotoxic exposure, and intra-arterial route. In at-risk patients, consider an alternative imaging method, start volume expansion, and utilize the lowest dose of contrast media consistent with a diagnostic result. Finally, determining eGFR 48–72 hours after receiving contrast media should be performed for CI-AKI detection.

Similarly, the KDIGO Clinical Practice Guidelines on Acute Kidney Injury [60] recommend that balancing the risk for CI-AKI against the benefit of administering contrast media should be firstly considered. Alternative imaging methods not requiring contrast media administration in patients at increased risk for CI-AKI so long as these yield the same diagnostic accuracy might be required. Before an intervention which encompasses a risk for CI-AKI, a baseline serum creatinine should be determined. Volume expansion with either isotonic sodium chloride or sodium bicarbonate solutions, rather than no volume expansion in patients at increased risk for CI-AKI, should be considered during hospitalization. In high-risk patients, a repeated serum creatinine should be performed at 12 and 72 hours after administration of contrast media. Prophylactic intermittent hemodialysis or hemofiltration did not have strong evidence in updated data for the purpose of CI-AKI prevention only.

In conclusion, contrast-induced AKI (CI-AKI) has been one of the leading causes for hospital-acquired AKI and is associated with independent risk for adverse clinical outcomes including morbidity and mortality. To prevent CI-AKI in patients who are receiving contrast media, every effort is required, including routine identification of at-risk patients, the use of appropriate hydration regimens, withdrawal of nephrotoxic drugs, selection of LOCM or IOCM, and using the minimum volume of contrast media as possible. There is no need to schedule the dialysis in relation to the injection of contrast media or the injection of contrast agent in relation to the dialysis program. Hemodialysis does not protect the poorly functioning kidney against CI-AKI.

## Figures and Tables

**Table 1:** Risk factors for contrast-induced acute kidney injury.

Patient-related	Procedure-related
(i) eGFR less than 60 mL/min/1.73 m^2^ before intra-arterial administration (ii) eGFR less than 45 mL/min/1.73 m^2^ before intravenous administration (iii) In particular, in combination with diabetic nephropathy dehydration congestive heart failure (NYHA grade 3-4) and low LVEF recent myocardial infarction (<24 hours) intra-aortic balloon pump periprocedural hypotension low hematocrit level age over 70 years concurrent administration of nephrotoxic drugs (iv) Known or suspected acute kidney injury	(i) Intra-arterial administration of contrast media (ii) High osmolality agents (iii) Large doses of contrast media (iv) Multiple contrast media administrations within a few days

**Table 2:** Summary of meta-analysis of randomized controlled trials to evaluate the benefit of sodium bicarbonate versus normal saline on prevention of contrast-induced acute kidney injury.

	Brar et al. (2009) [26]	Trivedi et al. (2010) [27]	Hoste et al. (2010) [28]	Kunadian et al. (2011) [29]	Jang et al. (2012) [30]
Inclusion study period	1966–November 2008	1950–May 2009	1950–February 20, 2009	Inception–September 2008	2001–January 31, 2012

Number of trials and included patients	14 trials (2,290 patients)	10 trials (1,090 patients)	18 trials (3,055 patients)	7 trials (1,734 patients)	19 trials (3,609 patients)

Setting and type of contrast	The majority of studies involved subjects undergoing cardiac angiography and using a nonionic low osmolar contrast	The majority of studies involved subjects undergoing cardiac angiography and using a nonionic low osmolar contrast	The majority of studies involved subjects undergoing cardiac angiography and using a nonionic low osmolar contrast	The majority of studies involved subjects undergoing cardiac angiography and using a nonionic low osmolar contrast	The majority of studies involved subjects undergoing cardiac angiography and using a nonionic low osmolar contrast

Results	(i) 3 trials were categorized as large (*n* = 1145) and 12 as small (*n* = 1145) trials. (ii) Among the large trials, the relative risk (RR) was 0.85 (95% CI 0.63 to 1.16) without evidence of heterogeneity. (iii) The pooled RR (95% CI) among the 12 small trials was 0.50 (95% CI 0.27 to 0.93) with significant between-trial heterogeneity. The small trials were more likely to be of lower methodological quality.	The use of sodium bicarbonate revealed an odds ratio (OR) of 0.57 (95% CI 0.38–0.85) for the occurrence of contrast-induced nephropathy.	The use of sodium bicarbonate demonstrated a benefit on the incidence of contrast-induced nephropathy (RR = 0.66, 95% CI 0.45–0.95).	The odds ratio (OR) for the development of contrast nephropathy for sodium bicarbonate versus sodium chloride was 0.33 (95% CI 0.16–0.69).	(i) Preprocedural hydration with sodium bicarbonate was associated with a significant decrease in the rate of CI-AKI (odds ratio [OR] 0.56; 95% CI 0.36–0.86). (ii) Stratified analyses by the type of contrast media suggested the use of sodium bicarbonate lower odds of CI-AKI in studies using low-osmolar contrast media (OR 0.40; 95% CI 0.23–0.71) compared with those using the isoosmolar agents (OR 0.76; 95% CI 0.41–1.43).

**Table 3:** Type of contrast media.

Type	Ionicity	Generic name	Iodine content (mg/mL)	Osmolality	Viscosity at 20–25°C (mPa·S)	Viscosity at 37°C (mPa·S)
HOCM	Ionic monomer	Diatrizoate	300–370	1500–2000	3.3–16.4	1.4–19.5
HOCM	Ionic monomer	Metrizoate	280–370	2100	5–9	2.8–5
HOCM	Ionic monomer	Iothalamate	141–480	600–2400	2–9	1.5–5.0
LOCM	Ionic dimer	Ioxaglate	280–320	600	12–15.7	6–7.5
LOCM	Nonionic monomer	Iohexol	140–350	322–844	2.3–20.4	1.5–10.4
LOCM	Nonionic monomer	Iopamidol	150–370	300–832	2.3–20.9	1.5–9.5
LOCM	Nonionic monomer	Iopromide	150–400	340–880	2.3–22	1.2–12.3
LOCM	Nonionic monomer	Iopentol	150–350	310–810	2.7–26.6	1.7–12.0
LOCM	Nonionic monomer	Iomeprol	150–400	301–730	1.9–27.5	1.4–12.6
IOCM	Nonionic dimer	Iodixanol	270–320	290	12.7–26.6	6.3–11.8
IOCM	Nonionic dimer	Iotrolan	240–300	270–320	6.8–16.4	3.9–8.1

HOCM: high osmolar contrast media, LOCM: low-osmolar contrast media, and IOCM: isoosmolar contrast media.

**Table 4:** Characteristics of the studies included in the meta-analysis to evaluate the benefit of dialysis versus routine preventive care on the incidence of contrast-induced acute kidney injury.

Authors	Year published	Study design	Radiocontrast agent	Technique	Timing	Duration of extracorporeal treatment (hour)	Dialyzer	Blood flow (mL/min)	Dialysate flow (mL/min)	RR for CI-AKI (95% CI)
Lehnert et al. [61]	1998	RCT	Iopentol	HD	63 ± 6 min after radiocontrast procedure	3	Fresenius F50 (Fresenius Medical Care, Bad Homburg, Germany)	139 ± 8	500	1.33 (95% CI 0.61–2.91)

Sterner et al. [62]	2000	RCT	Iohexol, Iodixanol, and Ioxaglate	HD	Maximum 3 hrs after radiocontrast procedure	4	Low-flux cellulose acetate or diacetate	200	500	1.70 (95% CI 0.59–4.90)

Vogt et al. [63]	2001	RCT	Nonionic low osmolarity	HD	Median 120 min (range 30–280) after radiocontrast procedure	3.1	Fresenius F50, F60 (Fresenius Medical Care)	180	500	1.27 (95% CI 0.80–2.01)

Berger et al. [64]	2001	RCT	Iopromide	HD	106 ± 25 min after radiocontrast procedure	2-3	Fresenius F6 (Fresenius Medical Care)	220	500	3.43 (95% CI 0.45–25.93)

Frank et al. [65]	2003	RCT	Iomeprol	HD	Simultaneously with radiocontrast procedure	4	Fresenius F60 (Fresenius Medical Care)	200	500	Total clearance of contrast media was significantly increased and the area under curve (AUC) of contrast media concentration was significantly lower in the HD group when compared with control group. However, the authors did not report incidence of CI-AKI.

Gabutti et al. [48]	2003	Prospective cohort	Ioversol	CVVHDF	Simultaneously with radiocontrast procedure	10	Prisma M100 (Prisma, Hospal, Mirandola, Italy)	150	Replacement and dialysate 2,000 mL/h	1.56 (95% CI 0.66–3.72)

Marenzi et al. [49]	2003	RCT	Iopentol	CVVH	Initiated 4–6 hrs before, interrupted during, and resumed after radiocontrast procedure	22–30	Renaflow HF700 (Gambro, Mirandola, Italy)	100	Replacement 1,000 mL/hour; no dialysate	0.12 (95% CI 0.05–0.32)

Hsieh et al. [66]	2005	Retrospective cohort	Iopromide	HD	As soon as technically feasible	4	AM-Bio HX90 (Asahi Medical Co, Ltd, Tokyo, Japan)	200	500	0.33 (95% CI 0.01–7.72)

Marenzi et al. [67]	2006	RCT	Iopental	CVVH	6 hours before and for 18 to 24 hours after contrast exposure (pre-/posthemofiltration group) 18 to 24 hours after contrast exposure (posthemofiltration group)	18–30	Renaflow HF700 (Gambro, Mirandola, Italy)	100	Replacement 1,000 mL/hour; no dialysate	0.05 (95% CI 0.01–0.41) (pre-/posthemofiltration group) 0.52 (95% CI 0.17–1.56) (posthemofiltration group)

Lee et al. [52]	2007	RCT	Iohexol	HD	As soon as technically feasible (81 ± 32 min, ranging from 45 to 180 min, after exposure to the contrast medium)	4	High-flux polysulfone membrane (BS1.8, Toray Industries, Inc., Tokyo, Japan).	150	500	0.07 (95% CI 0.01, 0.49)

Reinecke et al. [68]	2007	RCT	Iopromide	HD	Within 20 min after radiocontrast procedure	2	Fresenius 8, (Fresenius Medical Care)	180	500	Adjusted OR 2.2 (95% CI 0.9–5.7)

RCT: randomized controlled trial, HD: hemodialysis, CVVH: continuous venovenous hemofiltration, and CVVHDF: continuous venovenous hemodiafiltration.

**Table 5:** Nonpharmacological strategies to prevent contrast-induced acute kidney injury.

	Time of referral	Before the examination	Time of examination	After the examination
Elective examination	Identify patients who require measurement of renal function (i) Patients with known eGFR less than 60 mL/min/1.73 m^2^ determine eGFR (or SCr) after contrast medium administration within 7 days (ii) Patients who will receive intra-arterial contrast media (iii) Age over 70 years (iv) Patients with a history of renal disease renal surgery proteinuria diabetes mellitus hypertension gout (v) Recent nephrotoxic drugs	At-risk patients (i) Consider an alternative imaging method not using iodine-based contrast media (ii) Discuss the need to stop nephrotoxic drugs with the referring physician (iii) Start volume expansion. A suitable protocol is intravenous normal saline, 1.0–1.5 mL/kg/hour, for at least 6 hours before and after contrast media. An alternative protocol is intravenous sodium bicarbonate, 3 mL/kg/h for 1 hour before contrast medium and 1 mL/kg/hour for 6 hours after contrast media	At-risk patients (i) Use low- or isoosmolar contrast media (ii) Use the lowest dose of contrast media consistent with a diagnostic result	At-risk patients (i) Continue volume expansion (ii) Determine eGFR 48–72 hours after contrast media

Emergency examination	Identify at-risk patients (see Table 1) if possible (i) Determine eGFR if the procedure can be deferred until the result is available without harm to the patient (ii) If eGFR cannot be obtained, follow the protocols for patients with eGFR less than 60 mL/min/1.73 m^2^ for intra-arterial administration and eGFR less than 45 mL/min/1.73 m^2^ for intravenous administration as closely as clinical circumstances permit	At-risk patients (i) Consider an alternative imaging method not using iodine-based contrast media. (ii) Start volume expansion as early as possible before contrast media administration (see elective examination)	Patients not at increased risk Use the lowest dose of contrast media consistent with a diagnostic result	
